# Progress in Surgery

**Published:** 1899-06-24

**Authors:** 


### Progress in Surgery.

### GENERAL SURGERY OF THE ABDOMEN.

**Abdominal Wounds.**—A considerable advance has been made in the employment of transparent celloidin applications to wounds in the place of bulky absorbent dressings. By this means the fluids of repair are retained in the tissues, and not absorbed into dressings. Absolute sterility in operating is important, and the wound must be made quite dry and airless before applying the celloidin.[1] In closing abdominal wounds, Dr. Robert Reed advocates a fascia suture, which is passed after the interrupted sutures, and completed before they are tied.[2] Some after-effects of abdominal section have been traced by Miss May Thorne.[3] Of the cases of laparotomy two gave rise to ventral hernia as long as 14 years after operation, and one case of acute intestinal obtsruction occurred after a similar interval. Of 88 cases examined one returned for intestinal obstruction, and one for ventral hernia.

Occasionally remarkable recoveries are recorded from abdominal wounds. One such is that detailed of a woman disembowelled by a cow, most of the entrails resting outside the abdomen, including 20 ft. of the small gut. Four hours elapsed before reduction was effected, yet the patient made a good recovery.

With regard to gunshot injuries hope should be entertained even in desperate cases, as in that of Wölfler, recorded by Mannaberg.[5] In this there were 17 wounds of the small intestine, taking two and a half hours to sew up, yet the patient made a good recovery.

In some cases it is possible to diagnose and treat with success severe internal injuries when there has been no external wound, as in four recorded by Mr. Marmaduke Sheild and three by Mr. J. Hutchinson, jun.[6] Mr. Roughton has recently successfully treated a case of ruptured mesenteric artery.[7]

An interesting address was delivered by Mr. Charters Symonds before the Hunterian Society on the individual value of the symptoms in perforative peritonitis.[8] He draws attention to the following points:—

*Pain.*—This is common to the varieties of colic, to pleuro-pneumonia, and the like. It is the pain recurring after the period of repose which is a very grave sign, and hence should not be masked by sedatives. General abdominal distension and fixation are most important symptoms. Progressive distension, unrelieved by flatus tube, or turpentine enemata, is of grave significance. *Vomiting* is usually present in acute cases, and subsides with period of repose. It presses for operation in every case. It is the most reliable of all the symptoms.

*Temperature.*—This has no particular significance in strangulation. Temperature and pulse may steadily fall while dangerous septic processes are advancing.

*Period of Repose.*—This, if wrongly interpreted, may lead to disaster, and if deepened by sedatives will almost certainly do so.

*The General Condition.*—This may indicate grave suppura tion, although pulse and temperature be normal. Only experience can teach the value of these signs.

The advantages of drainage of the bowel in paralytic conditions, as in obstruction, are well known. In the case of peritonitis with much distension the same measure may be of use. Thus, Dr. Lilienthal has presented two such instances. He thinks as many incisions should be made as suffice to render the intestinal wall flaccid. In the discussion arising some surgeons gave it as their opinion that incisions were of little use, for they only relieved portions of the distended gut.

The abdomen is one of the favourite sites of "vanishing tumours." Attention has been drawn to these, from time to time, by Greig Smith, and others, and Mr. D'Arcy Power has added some recent examples.[9] Many of such cases, perhaps most of the abdominal ones, are of inflammatory origin, but the same cannot be said of "withering sarcomata" of the scalp, and of disappearing lymphangeiomata of the neck. Fæcal masses belong to a distinct category.

The anatomy of the peritoneal fossæ is of some importance to the surgeon, but its deliminations are somewhat overdone. Those who would desire intricate detailed description may refer to the recent lecture by Mr. Moynihan[10] on the subject.

Mesenteric tumours are of serious import owing to the difficulty of enucleating them without injuring the nutrition of the bowel and giving rise to gangrene and "fœtid diarrhœa." Bégouin and Herzog have tabulated a number of published cases. Of 36 fairly reported cases total extirpation was effected in 29, with only 14 recoveries.[11]

**Surgery of the Stomach.**—The successful removal of the entire stomach by Dr. Schlatter, of Zurich, is a triumph of surgical audacity. The patient lived for 14 months without the vestige of a stomach, and died from secondary deposits. From this it is obvious that adequate digestion will take place without a stomach.[12] Another case has been recorded by Dr. C. B. Brigham, of San Francisco.[13] It was reported six weeks afterwards. The union was effected by Murphy's button, and there appeared to be no impairment of digestive powers. Richardson also gives a case.[14] Union was here effected by Lembert's sutures, and the operation took an hour to perform. She is reported as enjoying life; her food satisfies her hunger, is digested perfectly well, and she is free from distress after taking food.

The use of Murphy's button is a great saving of time in gastro-intestinal surgery, although the results seem very uncertain. In Czerny's clinic gastro-enterostomy with Murphy's button has been performed in 83 cases with a mortality of 12·5 per cent.

There is an increasing tendency to explore the stomach as a diagnostic measure. The possibilities of this are summarised in a paper by Mr. Ernest Maylard.[15] Cases have been recorded by Bradford, Treves, and others of very thorough examination of the stomach, even to passing a light into the viscus, with cure of the patient, even though no lesion have been found. Maylard's case was one of plication of the stomach for dilatation; but it must be remembered that, although this surgeon advocates such explorations, the case given was nearly fatal from post-operative hæmorrhage.

Operations for perforated gastric ulcer have now become quite numerous and successful. Keen and Tinker have collected 78 cases in the last two years. Female cases were seven times more numerous than male ones, and the perforation was seven times as frequent on the anterior wall as on the posterior. The results are truly wonderful; the mortality has progressively diminished from 40 per cent. to 30 per cent., and lately to less than 20 per cent. Keen wisely adds "that when the profession at large understands that five patients out of six can be saved by immediate operation the mortality will still further diminish." Some cases lately published may be enumerated. One by Mr. Barling on a girl of 18 twelve hours after perforation. One by Mr. Thurston on a girl of 17 thirteen hours after perforation. Mr. Quarry Silcock relates an interesting case of recovery after perforation thirty-three hours previously, in which he used a gauze drain. Curious to relate, the patient got an attack of parotitis. The plausible suggestion is that these patients often suffer from stomatitis, and hence the salivary ducts are liable to infection from the mouth.[16] Mr. Robert Jones related two cases, one successful and the other living for fourteen days, ultimately dying from a foul perisplenic abscess. Mr. John Morgan has published two cases with one death, and Mr. Willoughby Furner three cases with one death. He suggests a high enterostomy in cases of threatened death from hæmorrhage from gastric ulcer. He also advocates an examination of the lesser peritoneal sac in every case, and if at all contaminated, a thorough flushing as well.

Operations for the effects of gastric ulcer are more recent than those for the ulcer itself. Mr. Tubby had a good case of arrest of hæmorrhage by including the ulcerating area in a peritoneal purse-string suture.[17]

With regard to resection of the stomach and pylorectomy for cancer, Kocher is of opinion that cancer of the stomach is in some cases a curable disease, and that patients may subsequently live for years.[18] Finney reports two cases of pylorectomy for cancer, one successful, and the other dying on the fifth day.

Mr. Rutherford Morrison brought before the Clinical Society a remarkably good series of gastric operation cases, including several pylorectomies for cancer. He thinks pylorectomy safer than gastro-jejunostomy.

[1] Med. Chron., April, 1899. [2] Med. Rec., Nov. 26, 1898. [3] Brit. Med. Jour., Feb. 4, 1899. [4] Lancet, Feb. 4, 1899. [5] Brit. Med. Jour., Feb. 18, 1899. [7] Brit. Med. Jour., Jan. 14, 1899. [8] Brit. Med. Jour., Mar. 4th, 1899. [9] Lancet, Mar. 4, 1899. [10] Lancet, Mar. 4, 1899. [11] Brit. Med. Jour., April 15, 1899. [12] New York Med. Rec., Mar 18, 1899. [13] Arnals of Surgery, Mar., 1899. [14] See Glasgow Med. Jour., Feb., 1899 [15] Lancet, April, 8, 1899. [16] Lancet, Mar. 25, 1899. [17] Practitioner, Dec. 1898. [18] Epit. Brit. Med. Jour., Dec. 31, 1898.

